# Long-term outcome of a dilated cardiomyopathy patient after mitral valve surgery combined with tissue-engineered myoblast sheets—report of a case

**DOI:** 10.1186/s40792-018-0549-6

**Published:** 2018-12-13

**Authors:** Shigeru Miyagawa, Keitaro Domae, Satoshi Kainuma, Ryouhei Matsuura, Daisuke Yoshioka, Hiroki Hata, Yasushi Yoshikawa, Koichi Toda, Yoshiki Sawa

**Affiliations:** Department of Cardiovascular Surgery, Osaka University Graduate School of Medicine, 2-2 Yamada-oka, Suita, Osaka 565-0871 Japan

**Keywords:** Cell, DCM, Tissue engineering, LVAD

## Abstract

**Background:**

Dilated cardiomyopathy (DCM) is a life-threatening heart muscle disease characterized by progressive heart failure, which often requires left ventricular assist device (LVAD) implantation or heart transplantation (HTx). A tissue engineering strategy using cell sheets for end-stage heart failure has emerged in recent years.

**Case presentation:**

Here, we describe a 50-year-old DCM patient with severe symptoms of heart failure with severe mitral regurgitation (MR) who was not a candidate for LVAD or HTx. The patient underwent mitral valve replacement followed by the transplantation of autologous myoblast sheets.

**Conclusion:**

The patient’s clinical symptoms improved with a preservation of cardiac performance, and he has survived for over 6 years since the combined surgery. This combined method was feasible and appeared to be effective, and thus represents a promising strategy for treating severe heart failure in end-stage DCM and as an alternative treatment for selected patients who are not candidates for LVAD or HTx.

## Background

Although left ventricular assist device (LVAD) implantation and heart transplantation which made great contributions to treatment for heart failure have been introduced to clinical situation with excellent clinical results, these surgical strategies have some limitations to treat end-stage heart failure such as durability of LVAD [1] and the donor shortage [2] especially in Japan. So this clinical situation has led physicians to consider alternative treatment for heart failure.

Conventional surgical strategy such as mitral valve surgery in dilated cardiomyopathy (DCM) patients with mitral regurgitation (MR) may have positive impacts on severe heart failure in selected patients [3]. But surgical intervention can treat only mitral valve not damaged myocardium, suggesting that additional treatment focused on damaged heart may be key point in successful surgical intervention for DCM with MR. Recent works concerning cell therapy have proposed functional amelioration in severe heart failure patients in clinical settings [4], proposing cell therapy may play adjuvant effect on mitral valve surgery for DCM patient with MR.

Here, we report a 50-year-old DCM patient with severe symptoms of heart failure with severe MR and treat by the combination of mitral valve replacement (MVR) and autologous myoblast sheet transplantation and have achieved long-term survival with functional preservation.

## Case presentation

Here, we report that a DCM patient with severe mitral regurgitation received MVR followed by the transplantation of autologous myoblast sheets, which had been manufactured in temperature-responsive culture dishes, and since then has survived for over 6 years with preserved cardiac performance and improved symptoms. The combined method was feasible for treating heart failure, and thus represents a potential strategy for heart failure patients with end-stage DCM who are not suitable for LVAD or HTx.

A 50-year-old man who suffered from idiopathic DCM had dyspnea on effort in 2000 and was emergently referred to a hospital. Ultrasonography revealed that the ejection fraction (EF) was 27%, MR grade was moderate, and tricuspid valve regurgitation grade (TR) was mild. Drug therapy including beta-blocker and ACE inhibitor was administered, but the symptoms did not improve. Instead, the patient was referred to the hospital several times due to recurrent heart failure. In 2011, he had a low-grade fever, poor appetite, and high T-bilirubin in the serum, and was admitted to Osaka University Hospital because of severe heart failure. Catecholamine infusion was started, his symptoms improved, and he was discharged from the hospital for several weeks. However, 3 months later, he was referred to the hospital again due to a recurrence in the heart failure.

Ultrasonography and right-heart catheter examination demonstrated severe heart failure [left ventricular diameter in diastole/systole (LVDd/Ds) = 83/75, EF = 31%, MR severe, TR moderate, pulmonary pressure (PAP) 62/28/41, pulmonary wedge pressure (PCWP) 28/44/30, right arterial pressure (RAP) 13, cardiac index (C.I.) 1.99]. Considering the patient’s severely distressed cardiac hemodynamics, he appeared to be a candidate for LVAD or HTx. However, he lacked familial support to maintain an implanted LVAD and therefore was not approved for LVAD or HTx. Ultrasonography and right-heart catheter examination indicated that the patient’s symptoms might have resulted from secondary pulmonary hypertension induced by severe MR. Because pulmonary hypertension can be lowered by mitral valve surgery, this procedure was planned to attempt to alleviate the patient’s symptoms.

In 2011, mitral valve (biological prosthetic valve) replacement (MVR) was performed without cardiac arrest, and the extracorporeal pump was successfully removed with an intra-aortic balloon pump (IABP) and adequate catecholamine infusion, and post-operative heart failure was well controlled with catecholamine infusion. The patient was weaned from the high dose of catecholamine without recurrent heart failure. He was then discharged from the hospital with a status of NYHA class II and free of catecholamine infusion. Six months after the operation, the patient’s exercise tolerance was well improved, with decreased pulmonary pressure and pulmonary wedge pressure, although the ejection fraction was severely reduced compared to its pre-operation value, because of damaged myocardium due to the DCM and the increased afterload after MVR. We therefore considered that an additional therapy to heal the damaged myocardium and preserve the cardiac structure or physiology might be effective for maintaining the patient’s cardiac performance.

**Fig. 1:**
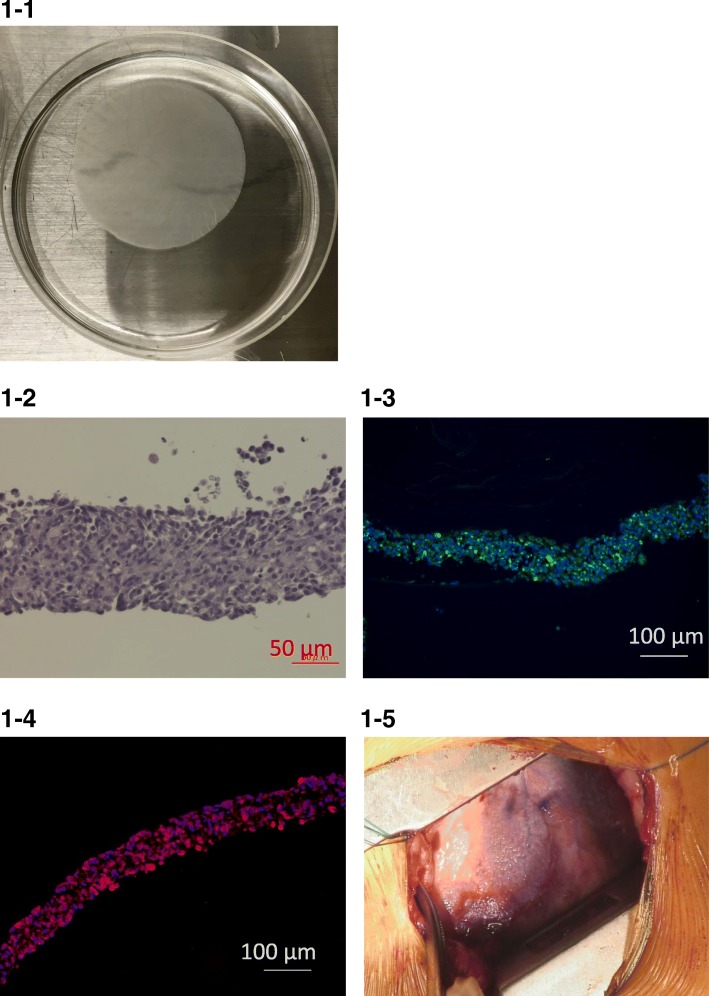
(1-1) The autologous myoblast sheets were round and about 5 cm in diameter. The purity of the myoblasts in the sheets was over 90%. (1-2) HE staining revealed a large number of cells connected to each other, with abundant extracellular matrix, in a cell sheet that was about 100-μm-thick. (1-3) Almost all of the cells in the cell sheet showed positive immunostaining for desmin in the cytosol. Green, desmin; blue, DAPI. (1-4) Almost all of the cells in the cell sheet showed positive immunostaining for actin in the cytosol. Red, actin; blue, DAPI. (1-5) Stacked autologous myoblast sheets were implanted onto six areas of the epicardium on the lateral and anterior LV wall through the fifth intercostal space

Myoblast cell sheet transplantation into human patients was reported to have angiogenetic, antifibrotic, and cell homing effects in a preclinical study approved by the Ethical Committee and Internal Review Board of Osaka University. Therefore, after the patient in the present case gave his informed consent, we conducted this procedure according to the Guidelines on Clinical Research Using Human Stem Cells from the Japanese Ministry of Health, Labour, and Welfare (UMIN ID; UMIN000003273; https://upload.umin.ac.jp/cgi-open-bin/ctr/ctr.cgi?function=brows&action=brows&recptno=R000003959&type=summary&language=J). An approximately 10-g piece of skeletal muscle was excised from the patient’s medial vastus muscle under general anesthesia. The autologous myoblasts derived from the skeletal muscle were then cultured according to published procedures, until 4 × 10^8^ cells were obtained (95% purity by FACS analysis with CD56). These cells were then seeded onto temperature-responsive culture dishes and formed cell sheets after 48 h, as previously reported (Fig. 1 (1-1)). Histological staining revealed that the cell sheet was approximately 100-μm-thick and that both desmin and actin were expressed in the cytosol of the cells (Fig. 1 (1-2, 1-3, 1-4)). Four myoblast sheets were then stacked and fixed with fibrin glue for ease in handling. In total, 24 autologous myoblast cell sheets were transplanted onto the anterior to lateral surface of the patient’s dilated heart, through a left lateral thoracotomy (Fig. 1 (1-5)).

**Fig. 2:**
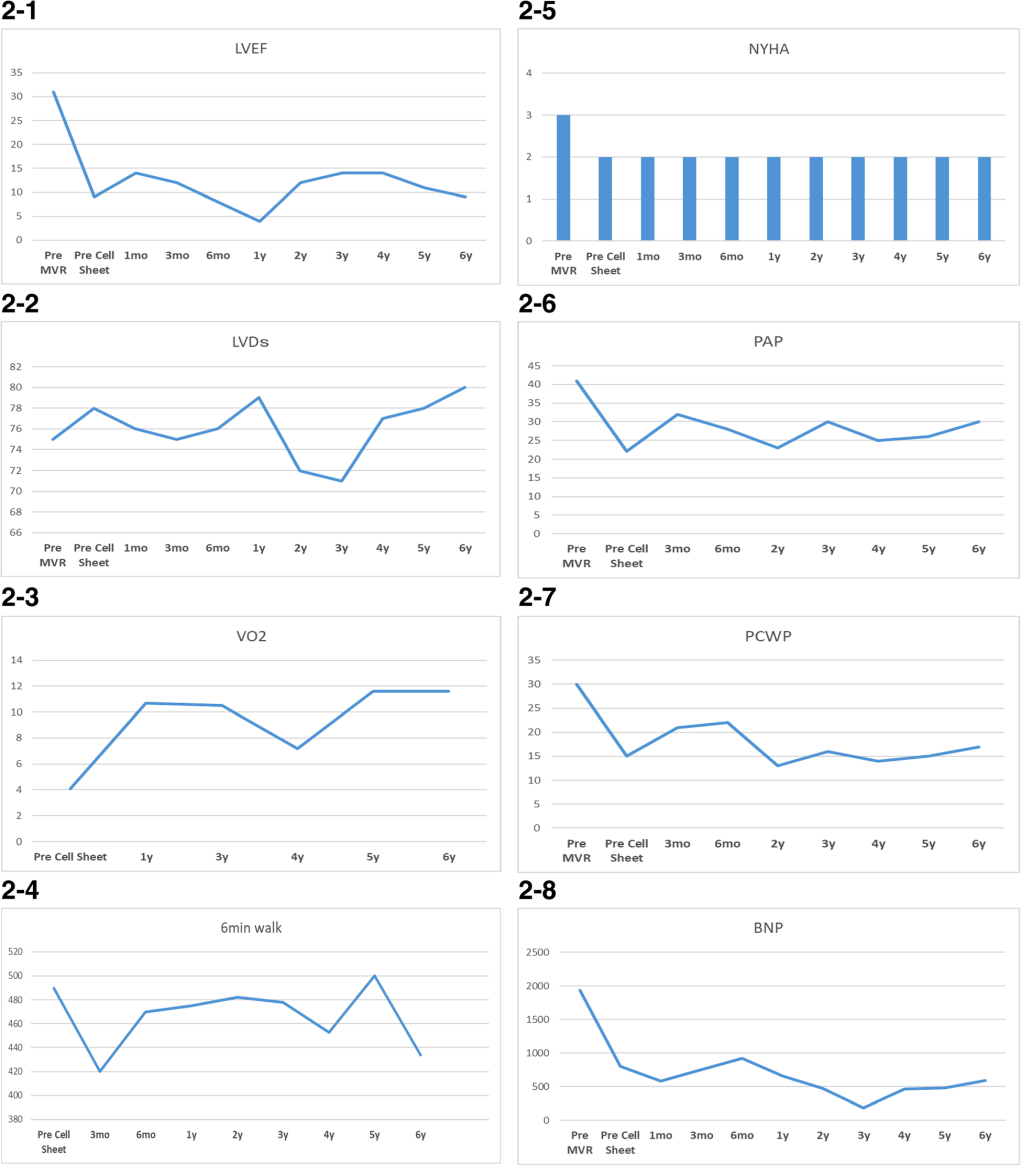
(2-1) Ultrasonography revealed that the EF declined after MVR and was preserved for 6 years after myoblast sheet transplantation. (2-2) Ultrasonography showed that the LVDs expanded after MVR and was generally preserved 6 years after myoblast sheet transplantation. In particular, 2 and 3 years after cell sheet transplantation, the LVDs were notably reduced, and the LVDs showed re-dilatation 4 years after cell sheet transplantation. (2-3) Exercise tolerance test revealed a notable elevation in the VO2 1 year after cell sheet transplantation, and this improvement was preserved for 6 years after transplantation compared with the pre-transplantation value. (2-4) A 6-min walk test demonstrated that the walking distance was preserved for 6 years after cell sheet transplantation compared with the pre-transplantation value, despite an initial drop 3 months after the operation. (2-5) The patient was NYHA class III before MVR, which was improved to class II. This improvement was preserved for 6 years after cell sheet transplantation. (2-6) After MVR, the PAP was notably decreased, and this improvement was maintained for 6 years after cell sheet transplantation. (2-7) After MVR, the PCWP was also notably improved, and this improvement was preserved for 6 years after cell sheet transplantation. (2-8) After MVR, the serum BNP was notably decreased, and it gradually improved after cell sheet transplantation and was preserved for 6 years

One month after myoblast sheet implantation, the patient was discharged without serious adverse events (SAE), and follow-up examinations revealed that the LV systolic function was improved after myoblast sheet implantation and preserved for 6 years after the operation (Fig. 2 (2-1)) and that the LVDs was maintained (Fig. 2 (2-2)) with preserved exercise tolerance (Fig. 2 (2-3, 2-4)) and symptoms (Fig. 2 (2-5)). In addition, the pulmonary pressure (Fig. 2 (2-6)) and pulmonary wedge pressure were well maintained after cell sheet implantation. Moreover, the serum BNP level was well maintained after the operation (Fig. 2 (2-8)). The actual patient survival was superior to the estimated survival rate calculated by the Seattle Heart Failure Model (1 year, 3 years, and 5 years = 60%, 37%, and 8%, respectively) [5], suggesting that this combined surgery prolonged the lifespan in this patient. We have not changed the dose of beta-blocker, diuretics, and ACE inhibitors and stop dosage of digitalis and pimobendan after combined surgery.

## Discussion

Because of the donor shortage for HTx [6] and the expense of LVAD, alternative strategies for heart failure are desired [7]. In this report, we reveal a possible new treatment for severe heart failure patients who are not candidates for implantable LVAD or HTx. The DCM patient described here underwent mitral valve surgery followed by myoblast sheet implantation. He showed improved clinical symptoms and has survived for over 6 years after the combined surgery, with preserved cardiac performance, exercise tolerance, and ameliorated pulmonary hypertension. However, further studies are needed to confirm whether the lifespan is prolonged after this combined therapy.

According to the recent guidelines for surgical options for heart failure [8], mitral valve surgery may be considered for severe heart failure patients, although the decision needs to be made with great care. While some studies report that some heart failure patients with severe MR show poor outcomes after mitral annuloplasty [9], others demonstrate feasible and reasonable outcomes [10], suggesting that the indication for mitral valve surgery is controversial. In this context, we previously reported that heart failure patients whose LV stroke work index is over 24 g/m/beat/m^2^ can show improved symptoms and preserved hemodynamics after this operation (unpublished data), suggesting that mitral valve surgery should be recommended for selected heart failure patients. In the patient reported here, the LV stroke work index was 11.3 g/m/beat/m^2^, which did not support an indication for mitral valve surgery according to our previous data. Nevertheless, we performed the surgical intervention to improve the patient’s symptoms by reducing the PAP, because he had a contraindication for LVAD. Further study may be needed to elucidate the appropriate criteria for mitral valve surgery in DCM patients with severe MR in addition to poor functional data.

In Japan and other countries, the number of heart transplants performed is quite small [11], and thus, clinicians have developed alternative strategies for saving heart failure patients. One such alternative strategy for heart failure patients with severe MR may be conventional surgery such as mitral valve surgery; this treatment may improve symptoms at lower cost and with fewer surgical complications compared with LVAD implantation or HTx. Therefore, if possible, conventional surgery should be considered for selected patients to improve symptoms or prolong survival. However, how to select the appropriate patients for conventional surgery is a critical issue that remains to be established.

One remaining question is whether MVR or mitral ring plasty (MAP) is better for severe heart failure patients. Although one report showed that the operative results were almost the same for MVR versus MAP for heart failure patients [12], we performed MVR for severe MR to avoid recurrent MR or SAE that might lead to readmission after surgery.

Severe heart failure is occasionally accompanied by MR, which is responsible for symptoms and an extremely damaged myocardium, worsening the progression of the disease. Some reports show that mitral valve surgery alone for MR does not reverse LV remodeling or preserve the LV geometry in heart failure patients who have a severely dilated LV chamber [3]. These findings suggest that additional therapy to treat the diseased myocardium may be required to prevent LV re-dilatation after surgery. Heart failure, especially due to DCM, features disease pathologies such as fibrosis, myocyte apoptosis, and ischemia [13], and a treatment target for DCM should focus on the damaged myocardium in addition to the valvular disease. Several recent papers reported that myoblast sheets applied to ischemic cardiomyopathy tissue [4, 14] could heal old myocardial infarction (OMI) [15, 16] or DCM [17, 18] in large or small animal models through potent angiogenesis, anti-fibrotic effects, and stem cell recruitment induced by cytokine paracrine effects. Here, we applied this innovative method to a patient in whom pulmonary hypertension was successfully controlled by MVR, to improve the damaged myocardium and maintain it after surgery.

The end point of this combined surgery in heart failure patients is not yet established. Although this treatment is unlikely to be superior to LVAD or HTx for obtaining higher perfusion in the organs, this combined therapy may be able to prolong the lifespan, improve symptoms, and reduce hospital readmissions, possibly by preserving both the systolic and diastolic LV performance. This combined therapy might have prolonged the lifespan of the patient in this case, as evidenced by his longer survival compared with the estimated survival rate calculated by the Seattle Heart Failure Model [5]. Thus, this combined therapy may provide benefits including longer survival, improved symptoms, preserved cardiac performance, and the avoidance of LVAD implantation in selected patients. Furthermore, in general, DCM shows progressive deteriorations in cardiac function and pathophysiology, so preserving the cardiac function or pathophysiology could have a great impact in treating DCM.

## Conclusions

In conclusion, anti-heart failure surgery combined with MVR and myoblast sheet implantation may be feasible for treating DCM with severe MR and may represent an alternative surgical strategy for treating severe heart failure that prolongs survival and improves symptoms. Promising results regarding the safety and functional recovery with this combined procedure warrant further clinical follow-up and larger studies to confirm its efficacy for DCM with severe MR.

## Abbreviations

BNP: Brain natriuretic peptide
C.I.: Cardiac index
DCM: Dilated cardiomyopathy
EF: Ejection fraction
HTx: Heart transplantation
IABP: Intra-aortic balloon pump
LVAD: Left ventricular assist device
LVDd/Ds: Left ventricle dimension in diastolic/dimension in systolic
MR: Mitral regurgitation
MVR: Mitral valve replacement
NYHA: New York heart association
OMI: Old myocardial infarction
PAP: Pulmonary pressure
PCWP: Pulmonary wedge pressure
RAP: Right arterial pressure
SAE: Serious adverse events
TR: Tricuspid valve regurgitation
